# Supplementary material for: Optical imaging of flavor order in flat band graphene
Source: Nat Commun. 2025 Jul 1;16:5555. doi: 10.1038/s41467-025-60675-8 (PMC12218313; doi:10.1038/s41467-025-60675-8)
Supplement: Supplementary file 1 — Supplementary Information [file 41467_2025_60675_MOESM1_ESM.pdf]

Supplementary Information for "Optical Imaging of Flavor Order in Flat Band Graphene"

Authors: Tian Xie[1], Tobias M. Wolf[2], Siyuan Xu[1], Zhiyuan Cui[1], Richen Xiong[1], Yunbo Ou[3], Patrick Hays[3], Ludwig F Holleis[1], Yi Guo[1], Owen I Sheekey[1], Caitlin Patterson[1], Trevor Arp[1], Kenji Watanabe[4], Takashi Taniguchi[5], Seth Ariel Tongay[3], Andrea F Young[1], Allan H. MacDonald[2]*, Chenhao Jin[1]*

Affiliations:

[1]Department of Physics, University of California at Santa Barbara, Santa Barbara, CA, 93116, USA

[2]Department of Physics, University of Texas at Austin, Austin, TX, 78712, USA

[3]Materials Science and Engineering Program, School of Engineering for Matter, Transport, and Energy, Arizona State University, Tempe, Arizona 85287, USA

[4]Research Center for Electronic and Optical Materials, National Institute for Materials Science, 1-1 Namiki, Tsukuba 305-0044, Japan

[5]Research Center for Materials Nanoarchitectonics, National Institute for Materials Science, 1-1 Namiki, Tsukuba 305-0044, Japan

* Corresponding author. Email: macd@physics.utexas.edu, jinchenhao@ucsb.edu

Contents:

Supplementary Note 1. Additional effects of dielectric screening

Supplementary Note 2. Comparison between moiré and non-moiré graphene

Supplementary Note 3. Sensitivity to flavor orders and transitions

Supplementary Note 4. Optical sensing at elevated temperatures

Supplementary Note 5. Effect of strain on exciton energy

Supplementary Note 6. Additional Information on Theoretical Methods

Supplementary Table 1. Tight-binding parameters (in eV) for rhombohedral trilayer graphene.

Supplementary Figure 1. More results on device D1.

Supplementary Figure 2. Extraction of 2s exciton energy.

Supplementary Figure 3. More results on device D2.

Supplementary Figure 4. More results on device D3.

Supplementary Figure 5. Inverse compressibility of device D3.

Supplementary Figure 6. Longitudinal resistance of device D5.

Supplementary Figure 7. Wide-field imaging of device D5.

Supplementary Figure 8. Local strain in WSe_2.

Supplementary Figure 9. Static polarizability of spin–valley polarized states in rhombohedral trilayer graphene (RTG)

Supplementary Figure 10. Static polarizability of the Landau-quantized electronic spectrum of bernal bilayer graphene at magnetic field B = 4 T

Supplementary Figure 11. Landau level spectra for bernal bilayer graphene (BBG) at large displacement fields.

Supplementary Figure 12. Phase diagram of bernal bilayer graphene with partially and fully polarized magnetic phases

Supplementary References

Suppelementary Note 1: Additional effects of dielectric screening

The effect of dielectric screening on 2s exciton binding energy is expected to be weak. Experimentally, it has been widely observed that 1s exciton energy in WSe_2 is much less sensitive to dielectric environment than 2s exciton[1]. Theoretically, the insensitivity of 1s exciton energy to dielectric screening is a consequence of the cancellation between quasi-particle bandgap shift and exciton binding energy change: a stronger screening will reduce both electron-electron and electron-hole Coulomb interaction, thereby reducing both the quasi-particle bandgap and the exciton binding energy. For 1s exciton, the two effects are comparable, and the quasi-particle bandgap shift is slightly larger. Therefore, stronger screening leads to a slight reduction (redshift) in the 1s exciton energy. In contrast, the 2s exciton shows a much larger redshift, indicating the dominant role of the quasi-particle bandgap shift. This can also be intuitively understood from the much smaller binding energy of the 2s exciton than the 1s exciton to start with. The screening-induced binding energy change of 2s exciton, therefore, is expected to be much smaller than that of 1s exciton and thus much smaller than the quasi-particle bandgap shift. The quasi-particle bandgap shift is dominated by the change of WSe_2 valance band. Since WSe_2 remains charge-neutral throughout our experiments, only the valence band is filled. The shift in the band gap can be viewed as originating from polaronic interactions between band-edge electrons in the semiconductor layer and charge fluctuations in the graphene layer, or equivalently from a change in the exchange contribution to the band gap (See supplementary materials).

We have used 2D Coulomb interaction in main text Eq. 1 instead of Rytova-Keldysh potential. Rytova-Keldysh potential has been widely used to account for the finite thickness of van der Waals layers, such as the deviation of exciton series in monolayer TMDC from an ideal 2D hydrogen model. On the other hand, main text Eq. 1 only involves intralayer interaction in graphene and interlayer interaction, where the Rytova-Keldysh potential is not suitable. In addition, the Rytova-Keldysh potential approaches the 2D Columb interaction at length scale much larger than the layer thickness, or momentum much smaller than inverse layer thickness. In our experiment, the relevant momentum is at most several k_F, much smaller than the inverse layer thickness of graphene.

Suppelementary Note 2: Comparison between moiré and non-moiré graphene

In MATBG and MATTG, we observe an additional replica feature at higher energy (Fig. 2a-c and Fig. 4b). Such replica features are discussed in detail in Ref.[2–4] and can be intuitively understood from a moiré potential in WSe_2 remotely imprinted from the graphene moiré superlattice. Such remote imprinting emerges in general TBG system away from magic angle (Fig. 4c) and is not related to the flavor physics. In contrast, the 2s exciton energy shift induced by flavor orders are consistently observed in all flatband graphene systems, regardless of whether a moiré pattern exists or not (Fig. 1-4 in the main text). This is expected since the 2s exciton energy shift is dominated by the quasi-particle bandgap shift, whose expression (main text Eq. 1) only involves graphene polarizability but not exciton wavefunction or moiré superlattices. Because flavor orders and transitions generally reconstruct the Fermi surface of graphene and change its polarizability, we expect our sensing technique to work equally well in moiré and non-moiré systems. Meanwhile, these flavor-induced features disappear in non-magic angle TBG (Fig. 4c), while both moiré superlattice and exciton replica still exist. Therefore, our observation is directly related to the flavor physics but not the moiré superlattice.

Experimentally, we find that the moiré graphene systems show more features in the optical sensing data than crystalline system, which is consistent with condition for flavor orders to emerge. One important distinction between the two categories is the origin of flatband. In the moiré case, such as MATBG and MATTG, flatband originates from the moiré superlattice and appear around “magic angles”, while a displacement field is not necessary[5,6]. In contrast, a large displacement field is required to make the bands flat in crystalline systems such as BBG and RTG[7,8]. Since flavor orders are intrinsically connected to the flatband, they typically require a finite D in crystalline systems but can appear at $D = 0$ in moiré systems[5,6]. Such behaviors have also been widely observed in experiments[9–14].

Suppelementary Note 3: Sensitivity to flavor orders and transitions

Our technique is most sensitive to phenomena that changes large-q part of graphene polarizability. Flavor orders and transitions generally belong to this category as they reconstruct the entire Fermi surface of graphene and change the polarizability up to several k_F. Examples include single-particle gaps induced by a small magnetic field, which shows strong signal in electrical measurements but much weaker in our optical sensing (Fig. 1 and 3 in the main text); A charge density wave (CDW) order typically also locally changes the Fermi surface, such as near the nesting vectors. In that case, the target layer polarizability will only change in a small range of q both around zero and around the nesting vectors. We therefore expect CDW of this type to show weaker signals in our sensing scheme compared to flavor orders, unless the CDW gap is very large.

Since flavor orders modify both small- and large-q part of graphene polarizability, they are expected to appear in both electrical measurements and our optical sensing. This, however, does not indicate the equivalence of the measurements. Compared to electrical measurements, our technique has two unique capabilities. First, it is more sensitive to large-q polarizability change and is less sensitive to phenomena that only involve small-q polarizability change. This allows us to distinguish between the two, especially when combined with electrical measurements. The comparison between flavor orders and Landau levels provides a good example, as discussed in the main text. For more quantitative comparison, we have performed measurements on the BBG/WSe_2 device D3 at a higher magnetic field of 7T (Extended Fig. R4g-i). The Landau gap features are clearly visible (red arrows), but much weaker than the features within the zeroth Landau level. We can also compare the features in MATBG at integer fillings $-3 \leq v \leq 3$ to the features at v=± 4. The latter shows orders-of-magnitude stronger features in both electrical transport and compressibility measurements than the former owing to the much larger single-particle gaps[15–19]. In contrast, the optical features at v=± 4 is at most comparable, and often weaker than those at $-3 \leq v \leq 3$ (Fig. 4b). These behaviors are also observed in other studies[2,20].

Second, our technique is sensitive to phenomena that *only* changes finite-q polarizability, which cannot be detected by electrical measurement. For example, we observe prominent redshifts of exciton energy at odd Landau level fillings in BBG/WSe_2 (Fig. 3); while electrical measurements only show features at even fillings. This can be naturally understood from orbital polarization at odd fillings within the zeroth Landau level, which enhances the finite-q polarizability of graphene. Such physics can be uniquely accessed with our sensing techniques.

Supplementary Note 4: Optical sensing at elevated temperatures

Experimental probes of isospins orders in graphene can be divided into two types. The first type requires a single domain, such as Hall and SQUID measurements of magnetization. Two domains with opposite magnetization would give opposite signal in SQUID that cancels each other. In contrast, the second type does not require a single domain, such as chemical potential and compressibility measurements[21]. Our optical sensing belongs to the second type: the signal originates from dielectric screening, which is insensitive to the sign of order parameter. For example, two domains with opposite spins would give the same shape of Fermi surface (despite from different spins) and therefore the same dielectric function. Consequently, the existence of multiple domains does not affect the second type of measurements as long as the domains size is much larger than $1/k_F$ and the domain lifetime much larger than $1/E_F$, such that the Fermi surface is well defined.

When the temperature exceeds the critical temperature, long-range flavor order vanishes. On the other hand, there can still be short-range orders or strong fluctuations in the order parameters. They can be intuitively understood as spontaneously formed domains that keep fluctuating over space and time. Their signals would disappear in the first type of measurement but remain finite in the second type until the characteristic length/timescale of the fluctuating orders drop below the threshold above. In addition, since the large-q part of polarizability is less sensitive to a finite domain size, we expect our optical sensing technique to be able to capture shorter-range orders and weaker fluctuations compared to zero-q measurements such as compressibility.

Supplementary Note 5: Effect of strain on exciton energy

In our flatband graphene/WSe_2 devices, strain can accumulate in both graphene and WSe_2 layers during the fabrication process. Local strain in WSe_2 will not affect our sensing technique. As has been widely reported[22–24], the main effect of local strain in monolayer 2D semiconductors is shifting the bandgap and exciton resonances. On the other hand, the detection of flavor orders in our optical sensing technique relies on the relative exciton energy shift induced by charges in graphene. Because WSe_2 remains charge neutral throughout the measurement, any local strain in WSe_2 will only generate a constant background in the exciton energy that does not depend on graphene carrier concentration.

The different 2s exciton energies in Figure 2-4 do not indicate local strain. Instead, this is naturally expected from different dielectric screening. Even when graphene layers are charge-neutral, they still provide a large dielectric screening (e.g. compared to hBN). Such screening depends sensitively on the band structure of graphene, thereby varying significantly between different graphene systems. We have also directly calibrated the local strain in WSe_2 from the exciton resonance shifts at graphene charge neutrality. Supplementary Fig.8 shows the 1s exciton resonance of MATBG/WSe_2 device D5 at representative spatial spots labelled in Supplementary Fig.8. The 1s exciton energy only varies slightly (<5meV) throughout the sample, indicating a small local strain of <0.1%[24].

On the other hand, strain in graphene is an important source of disorder, which will affect the correlated physics and therefore our measurement results. It is known that the behavior of MATBG varies significantly over devices even with similar twist angles due to the local strain. For example, the lattice relaxation and domain formation in TBG is reported to dramatically change the band structure and correlated physics under a fixed twist angle[25]. Strains of different symmetry also have significant effects on the preferred ground states in MATBG[26]. These effects are key to understanding correlated physics in flatband graphene systems and are under intensive investigations. The high throughput, wide field imaging capability of our sensing technique opens up opportunities in studying the spatial variance of correlated orders and elucidating the effects from local strain.

Supplementary Note 6: Additional Information on Theoretical Methods

Here we provide supplemental information summarizing our theoretical models for (i) the valence band exchange correction due to proximate polarizable layers, (ii) the polarizability of rhombohedral trilayer graphene at $B_z = 0$ obtained from band structure calculation, (iii) the polarizability of rhombohedral bilayer graphene at finite B fields accounting for Landau quantization.

Exchange Correction to the Semiconductor Layer Band Gap

The dielectric environment surrounding a single-layer TMD, such as WSe_2, can strongly renormalize both the exciton binding energies and the single-particle band gap[1,27,28]. While for 1s excitons, the two effects are observed to be of the same order of magnitude (and thus cancel approximately in the resonance energy), for ns excitons with $n \geq 2$, the effect of band gap renormalization is systematically observed to be dominant. The present work aims to highlight how this band gap shift in a TMD monolayer (the "sensing layer") can be used to witness electronic properties of a nearby target layer, and in particular highlights that this probe is sensitive to layer and flavor polarizdation.

When the charge response in the nearby layer is rapid and the change in the interaction potential is almost instantaneous, the leading order effect that changes the band gap $E_{gap} \rightarrow E_{gap} + \delta E_{gap}$ is a reduction in the exchange contribution to the band gap:

$$\delta E_{vb} \simeq -\int \frac{d^2q}{(2\pi)^2}\left[W_{11}(\boldsymbol{q}, \omega = |\epsilon_{\boldsymbol{q}}|) - V_{11}(\boldsymbol{q})\right] \quad (1)$$

Where W_{11} is the retarded screened interaction between charge carriers within the insulating sensing layer (layer 1) in the presence of the probed layer (layer 2), and V_{11} is the interaction without it. In supplementary Eq. (1) we have made the safe assumption that the change in the interaction potential is smooth on the semiconductor layer lattice constant length scale, which allows the valence band exchange energy to be evaluated using an electron gas formula. The shift in the band gap can equivalently be viewed[29] as arising from polaronic shifts in the conduction band minimum and valence band maximum energies due to interactions with charge fluctuations in the probed layer. In the following sections, we will discuss how we model the screened interaction, and evaluate it for examples relevant to the experiments in the main text.

Screened Interaction Within the Sensing Layer

The retarded screened intralayer-interaction $W_{11}(\boldsymbol{q}, \omega)$ contains contributions induced by the proper polarizability $\Pi_{22}(\boldsymbol{q}, \omega)$ of the target layer. Explicitly $W(\boldsymbol{q}, \omega) = \epsilon(\boldsymbol{q}, \omega)^{-1} V(q)$, where the dielectric response is related to the bare interactions V and the polarizability Π through $\epsilon(\boldsymbol{q}, \omega) = 1 - V(q)\Pi(\boldsymbol{q}, \omega)$. Assuming that Π is dominated by contributions from the target layer, we find

$$W_{11}(\boldsymbol{q}, \omega) \approx V_{11}(q) + \chi_{22}(\boldsymbol{q}, \omega) V_D(\boldsymbol{q}, \omega)^2 \qquad , \chi_{22}(\boldsymbol{q}, \omega) = \frac{\Pi_{22}(\boldsymbol{q}, \omega)}{1 - \mathrm{V}_{22}(\boldsymbol{q})\Pi_{22}(\boldsymbol{q}, \omega)} \quad (2)$$

where χ_{22} of the charge response in the target layer (2) and intra- and interlayer interactions are

$$V(\boldsymbol{q}) = \begin{pmatrix} V_{11}(\boldsymbol{q}) & V_D(\boldsymbol{q}) \\ V_D(\boldsymbol{q}) & V_{22}(\boldsymbol{q}) \end{pmatrix}, \quad V_D(\boldsymbol{q}) = \frac{2\pi e^2}{\epsilon_r' \, \boldsymbol{q}} e^{-qd}, \quad V_{22}(\boldsymbol{q}) = \frac{2\pi e^2}{\epsilon_r \, \boldsymbol{q}} \qquad (3)$$

We note that the TMD monolayer intralayer interaction $V_{11}(\boldsymbol{q})$, usually described as effective Rytova-Keldysh potential[30], is not relevant for the relative shift described by supplementary Eq. (1). The interlayer distance d is about $d \sim 2a_G$ without a spacer and about $d \sim 20a_G$ with a spacer. For the dielectric constants, for simplicity, we assume $\epsilon_r' \simeq \epsilon_r \simeq 5$ without affecting our results significantly. It is worth highlighting, that here we assume that the sensing layer interacts with a single target layer. If needed, we could straightforwardly extend this model to account for additional layer degrees of freedom in V and Π, but we will instead focus on qualitative aspects unrelated to layer polarization in the target system.

In the random-phase approximation (RPA), the polarizability $\Pi_{22}(\boldsymbol{q}, \omega)$ in the target layer is just the bare polarization bubble diagram. In terms of the non-interacting single-particle eigenbasis, we have

$$\Pi_{22}(\boldsymbol{q}, \omega) = \frac{1}{A_{uc}} \sum_{n,m,\alpha,\boldsymbol{k}} \frac{f(\varepsilon_{n\alpha}(\boldsymbol{k})) - f(\varepsilon_{n\alpha}(\boldsymbol{k}+\boldsymbol{q}))}{\varepsilon_{n\alpha}(\boldsymbol{k}) - \varepsilon_{n\alpha}(\boldsymbol{k}+\boldsymbol{q}) + \omega + i\delta} \times |\langle \psi_{n\alpha}(\boldsymbol{k}) | \psi_{m\alpha}(\boldsymbol{k}+\boldsymbol{q}) \rangle|^2 \qquad (4)$$

where $\varepsilon_{n\alpha}(\boldsymbol{k})$ is the energy band n for orbital index α, and $|\psi_{m\alpha}(\boldsymbol{k})\rangle$ the corresponding wavefunctions, and A_{uc} is the unit cell area. The overlap matrix element is often referred to as form factor. Similarly, in presence of a static homogeneous transverse magnetic field $\boldsymbol{B} = B\hat{z}$, the polarizability in terms of the Landau level spectrum is[31]

$$\Pi_{22}(\boldsymbol{q}, \omega) = \frac{1}{2\pi l^2} \sum_{n\alpha, n'\alpha'} \frac{\Theta(\epsilon_F - \varepsilon_{n\alpha}) - \Theta(\epsilon_F - \varepsilon_{n'\alpha'})}{\varepsilon_{n\alpha} - \varepsilon_{n'\alpha'} + \omega + i\delta} \left|\mathcal{F}_{n\alpha,n'\alpha'}(\boldsymbol{q})\right|^2 \qquad (5)$$

where $\varepsilon_{n\alpha}$ are the Landau levels for integer n and orbital α, and $\mathcal{F}_{n\alpha,n'\alpha'}(\boldsymbol{q})$ are multiorbital magnetic form factors, which are given by orbital superpositions of the magnetic form factors of the two-dimensional electron gas.

In supplementary Fig. 9, we show the static polarizabilities for flavor-polarized states in rhombohedral trilayer graphene in a large displacement field, and in supplementary Fig. 10 we show the static polarizability of bernal bilayer graphene in a strong magnetic field at filling factors corresponding to the nearly-orbital-degenerate lowest two Landau levels. In both cases, we find that spontaneous polarization of electronic states in the graphene material should lead to significant band gap renormalization in a nearby WSe_2 sensing layer, in agreement with experiment observations.

<u>Rhombohedral-Stacked Multilayer Graphene</u>

In what follows, we will briefly review the band structure model and Landau quantization for the rhombohedral multilayer graphene that we employ to evaluate polarizabilities in

supplementary Eqs. (4) and (5). In giving explicit expressions, we will focus on the trilayer case - the bilayer case requires minor changes to the basis and to the tight-binding parameters.

Continuum model. Rhombohedral trilayer graphene (RTG) has a multi-layered triangular lattice structure with lattice constant $a = 2.46$Å and 6 atoms per unit cell: three layers (separated by $d = 3.4$Å), each with two sublattices. Neighboring layers and next-neighboring layers are mutually AB-stacked. We employ the commonly-used and well-known continuum model for the low-energy dispersion of π-electrons with spin $s = \pm 1/2$ near each nonequivalent valley τK with $\tau = \pm 1$ at Brillouin zone corners[32,33]. We label the four spin-valley combinations as flavor index $\alpha = (s, \tau)$. Ordering the basis as A1, B1, A2, B2, …, the resulting continuum Hamiltonian per flavor is[32]

$$h(\boldsymbol{k}) = \begin{bmatrix} t(\boldsymbol{k}) + U_1 & t_{12}(\boldsymbol{k}) & t_{13} \\ t_{12}^{\dagger}(\boldsymbol{k}) & t(\boldsymbol{k}) + U_2 & t_{12}(\boldsymbol{k}) \\ t_{13}^{\dagger} & t_{12}^{\dagger}(\boldsymbol{k}) & t(\boldsymbol{k}) + U_3 \end{bmatrix}_{6\times 6} \quad (6)$$

where $\boldsymbol{k} = (k_x, k_y)$ is the Bloch momentum measured w.r.t.~valley τ. The model contains intralayer hopping (t), nearest-layer hopping (t_{12}), and next-nearest-layer hopping (t_{13}):

$$t(\boldsymbol{k}) = \begin{bmatrix} 0 & v_0\pi^{\dagger} \\ v_0\pi & 0 \end{bmatrix}, \quad t_{12}(\boldsymbol{k}) = \begin{bmatrix} -v_4\pi^{\dagger} & v_3\pi \\ \gamma_1 & -v_4\pi^{\dagger} \end{bmatrix}, \quad t_{13}(\boldsymbol{k}) = \begin{bmatrix} 0 & \gamma_2/2 \\ 0 & 0 \end{bmatrix} \quad (7)$$

where $\pi = \tau k_x + i k_y$ is the linear momentum, and γ_i ($i = 0, \dots, 4$) are tight-binding parameters with corresponding velocity parameters $v_i = (\sqrt{3}/2) a\gamma_i/\hbar$. We use the model parameters listed in supplementary table 1, which are chosen to match quantum oscillation frequency signatures in Ref.[13]. We include different layer potentials (through the U_i-terms) induced by top and bottom gate, to correclty account for the electric displacement field D and the electronic density n_e.

We note that intrinsic Ising-type spin-orbit coupling (SOC) λ is negligible (about 10-50 μeV), but proximity to transition metal dichalcogenides (e.g., WSe_2 without spacer) can drastically enhance it in the nearest graphene layer (up to $\lambda \sim 0.800$ meV). The impact of proximity-induced SOC on the electronic bands is highly sensitive to the sign of the displacement field D. In this work, we neglect SOC to focus on qualitative effects and leave the analysis of SOC for future studies.

Landau quantization. Transverse magnetic fields quantize the electronic spectrum into Landau levels, described by a minimal coupling $\boldsymbol{p} \mapsto \pi = \boldsymbol{p} - e\boldsymbol{A}$\$ in supplementary Eq. (6). The commutation relation of $\pi = \pi_x + i\pi_y$ with $\pi^{\dagger}$ implies ladder operators $\hat{a}$ and $\hat{a}^{\dagger}$ with $[\hat{a}, \hat{a}^{\dagger}] = 1$ such that $\pi_x = \frac{\hbar}{\sqrt{2} l_B}(\hat{a}^{\dagger} + \hat{a})$, $\pi_y = \frac{\hbar}{\sqrt{2} l_B}(\hat{a}^{\dagger} - \hat{a})$. We defined the magnetic length $l_B = \sqrt{\hbar/eB}$. Each Landau level has degeneracy Φ/ϕ_0, where $\Phi = BA$ is the flux and $\phi_0 = h/e$ a flux quantum, and thus accommodates the electronic density $n_B =$

$1/(2\pi l_B^2)$. Writing the Hamiltonian in the eigenbasis of $\hat{N} = \hat{a}^\dagger \hat{a}$ allows to obtain the electronic spectrum (numerically and in simplified cases analytically), see supplementary Fig. 11 for the case of bernal bilayer graphene (BBG).

Flavor magnetism. Experiments studying top/back-gated rhombohedral multilayers demonstrate that correlated states such as spin-valley magnetism, superconductivity and fractional anomalous quantum Hall states are ubiquitous in these materials[11–14,34–36]. The simple Coulomb potential commonly used to approximate effective intralayer interactions is $V_q = \left(\frac{2\pi k_e}{\epsilon_r}\right) \tanh(|\boldsymbol{q}| d_{gate}) / |\boldsymbol{q}|$, for metallic gates at distance $d_{gate} \in [30,60]$ nm, effective relative permittivity $\epsilon_r \in [7,20]$, and Coulomb constant k_e=1.44eV nm. The spin-valley magnetism can (to a limited degree) be understood from a mean-field perspective: the energy gain in exchange energy by sequentially occupying individual flavors can outweigh the cost associated with the kinetic band energy, especially if the band dispersion is flat/the density of states is large. In supplementary Fig. 12, we illustrating this effect by showing the total energy of different spin-valley polarized states obtained from self-consistent Hartree-Fock mean-field calculations for the case of bernal bilayer graphene.

Supplementary Table

γ_0	γ_1	γ_2	γ_3	γ_4	U	Δ	δ
3.160	0.380	-0.015	-0.290	0.141	0.030	-0.0023	-0.0105

Supplementary Table 1. Tight-binding parameters (in eV) for rhombohedral trilayer graphene, see also Refs. [13,32,33]

Supplementary Figures

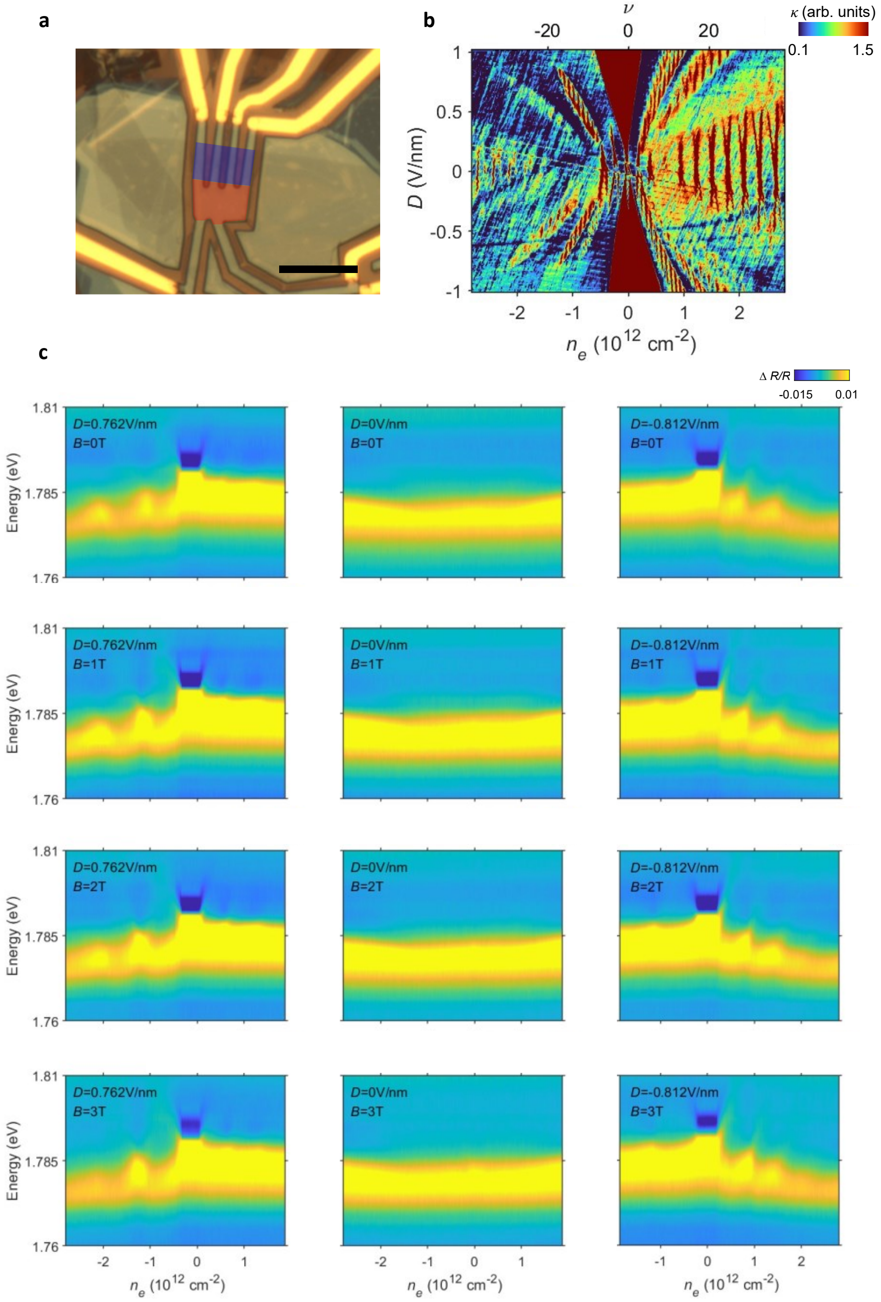

Supplementary Figure 1. More results on device D1. (**a**) Optical microscope image of RTG device D1. Scale bar: 10μm. It has two regions with (red) and without (blue) WSe_2, respectively, giving rise to two slightly offset patterns in compressibility (Fig. 1b and (**b**)). On the other hand, RC is not affected by such spatial inhomogeneity since it is a local measurement. (**b**) Displacement-field and carrier-density dependent inverse compressibility under magnetic field *B*=3T. Prominent incompressible peaks emerge from Landau gaps. (**c**) RC at representative displacement fields *D*=0.762, 0, and -0.812V/nm under magnetic field *B*=0 to 3T. No features correspond to Landau gaps are observed. All measurements are performed at 3K.

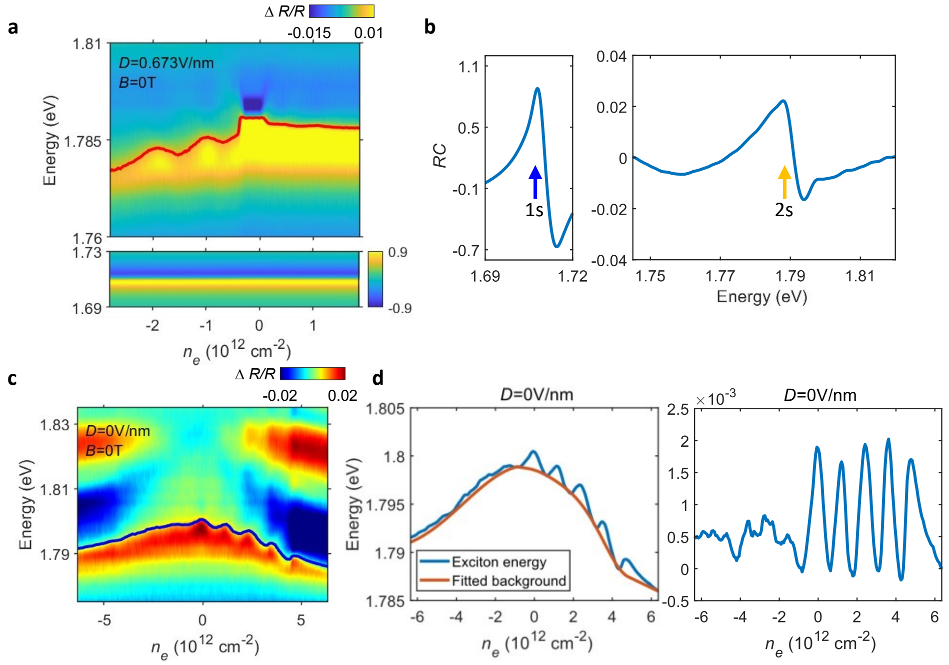

Supplementary Figure 2. Extraction of 2s exciton energy. (**a**) Upper panel: RC of device D1 at D=0.673V/nm with the extracted 2s exciton energy overlaid on top (red line). Lower panel: the RC near 1s exciton resonance, which remains largely unchanged over doping. (**b**) Representative RC spectrum of device D1 at D=0.673V/nm and n_e=0cm^{-2}. The 1s and 2s exciton resonances are marked by blue and yellow arrows, respectively. (**c**) RC of device D2 at D=0V/nm with the extracted 2s exciton energy overlaid on top (blue line). (**d**) Left: The extracted 2s exciton energy (blue) and the fitted background (orange). Right: background-subtracted 2s exciton energy showing clear peaks from the cascade features.

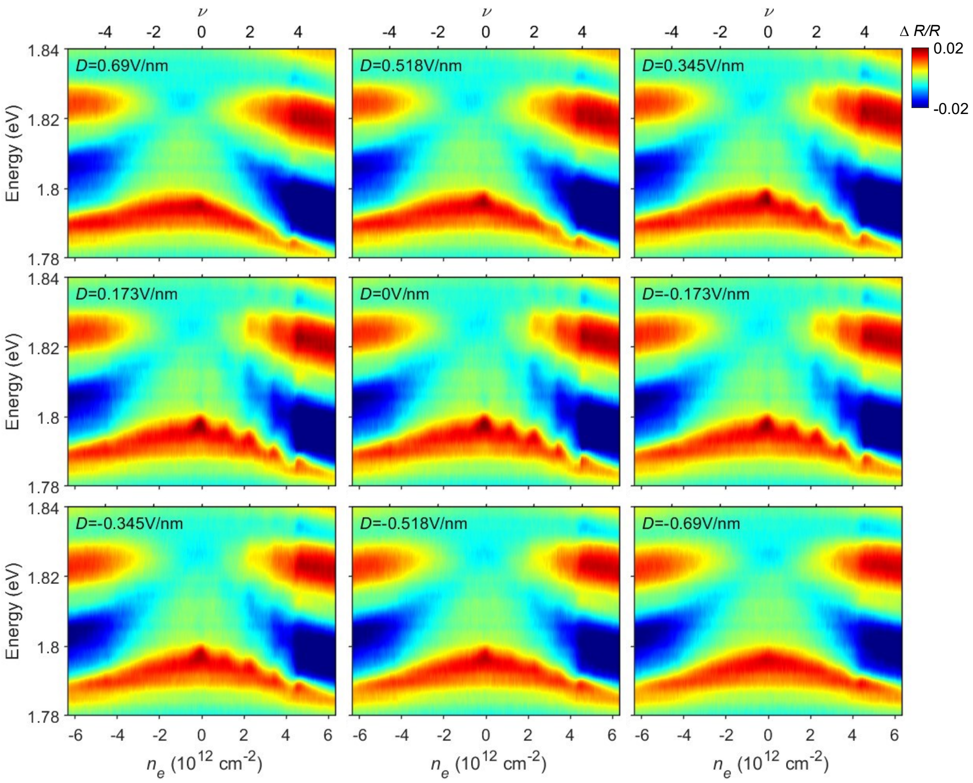

Supplementary Figure 3. More results on device D2. RC of MATTG device D2 at $D = \pm0.69$, ±0.518, ±0.345, ±0.173, and 0V/nm. The cascade features at integer filling become weaker at larger displacement field. All measurements are performed at 3K.

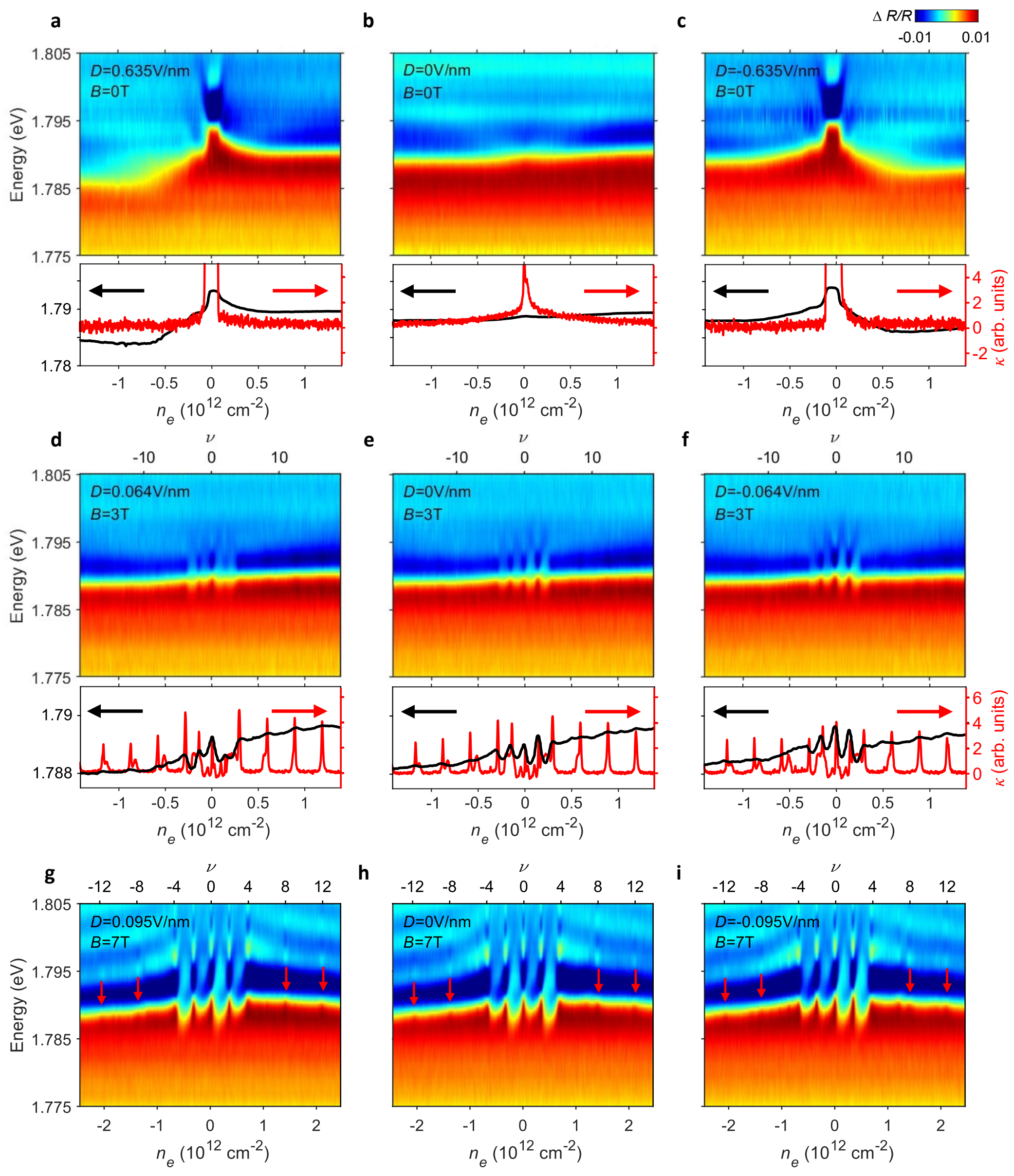

Supplementary Figure 4. More results on device D3. (**a**)-(**c**) Upper panel: RC of BBG device D3 at B=0T and D = 0.635 (**a**), 0 (**b**), and -0.635V/nm (**c**). Lower panel: extracted 2s exciton energy (black) and comparison with inverse compressibility (red). (**d**)-(**f**) Upper panel: RC of device D3 at B=3T and D = 0.064 (**d**), 0 (**e**), and -0.064V/nm (**f**). Lower panel: extracted 2s exciton energy (black) and comparison with inverse compressibility (red). The 2s exciton energy shows prominent oscillations within the zeroth Landau level. (**g**)-(**i**) RC at B=7T and D = 0.095 (**g**), 0 (**h**), and -0.095V/nm (**i**). Red arrows label Landau gaps outside the zeroth Landau level.

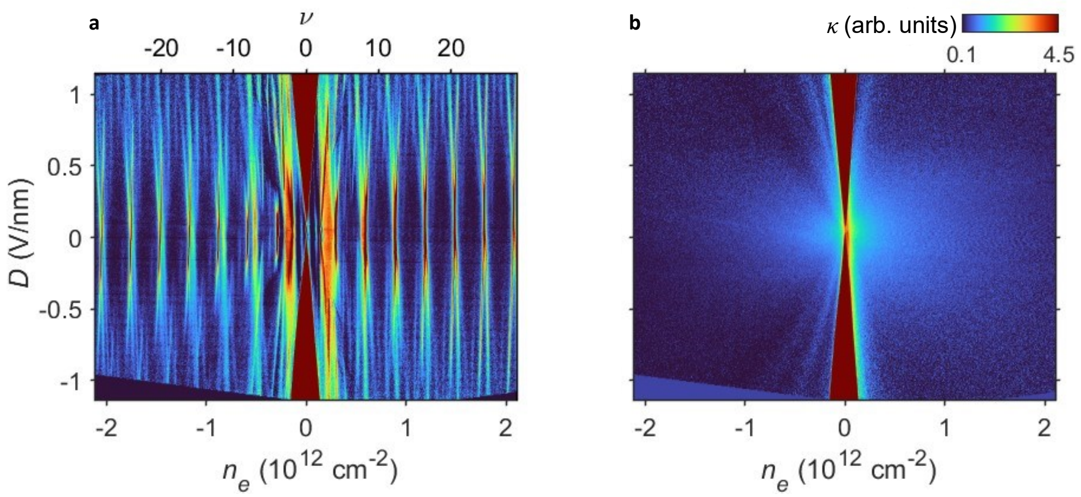

Supplementary Figure 5. Inverse compressibility of device D3. (**a**)(**b**) Inverse compressibility as a function of displacement field and carrier density at B= 3 (**a**) and 0T (**b**).

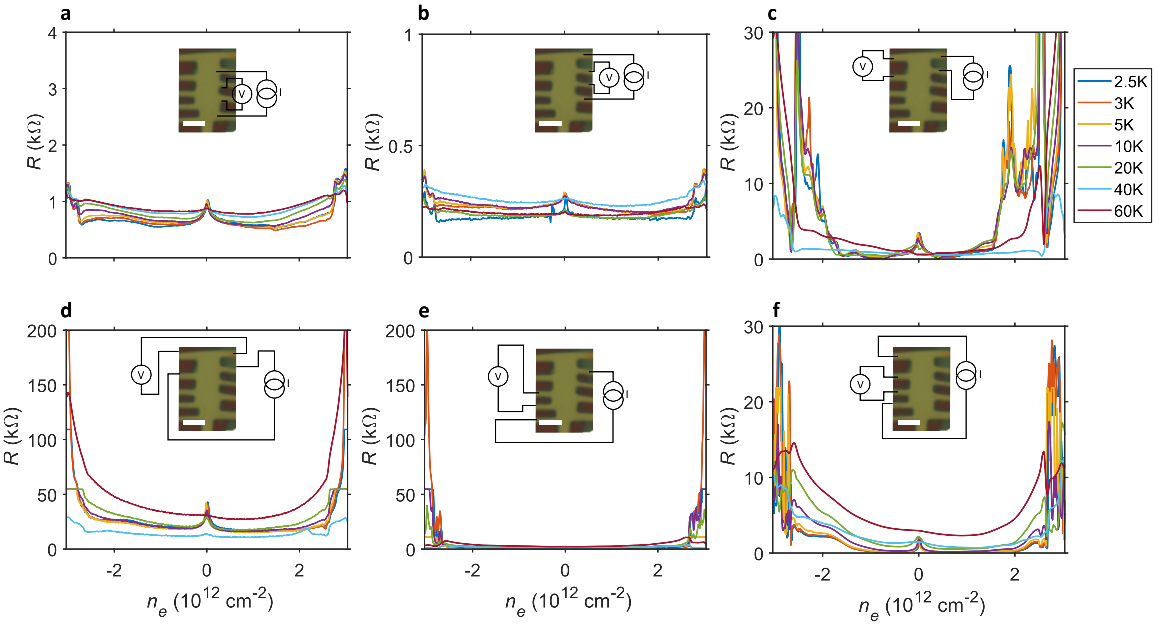

Supplementary Figure 6. Longitudinal resistance of device D5. (**a**)-(**f**) Temperature and carrier-density dependent longitudinal resistance of device D5 using different terminal configurations. The insulating feature at ν=3 only appears in measurement configuration (**c**), consistent with the RC measurement. Scale bar: 3μm

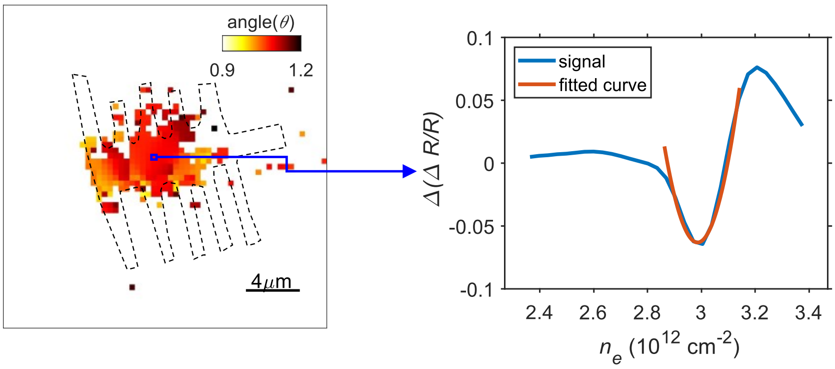

Supplementary Figure 7. Wide-field imaging of device D5. Left: spatial map of twist angle extracted from the $v = 4$ cascade feature. Right: carrier-density dependent differential reflection (blue) near $v = 4$ for the spatial spot marked by the blue box. The dip associated with cascade feature is fitted by a 2nd-order polynomial, as detailed in the methods. The extracted carrier density is $n_{v=4} \approx 2.95\times10^{12}$cm^{-2}, corresponding to 1.12° twist angle.

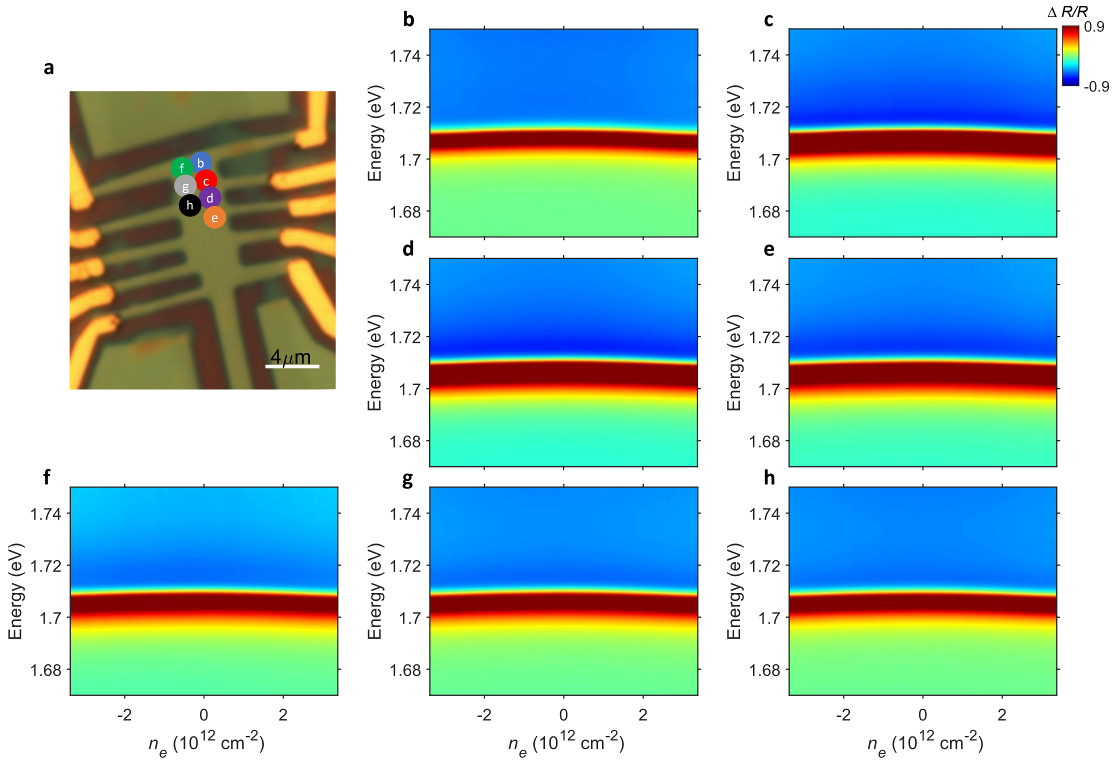

Supplementary Figure 8. Local strain in WSe₂. (**a**) Optical microscope image for MATBG device D5. Scale bar: 4μm. (**b**)-(**h**) RC of different spots in device D5 around 1s exciton resonance. Their locations are marked in (**a**).

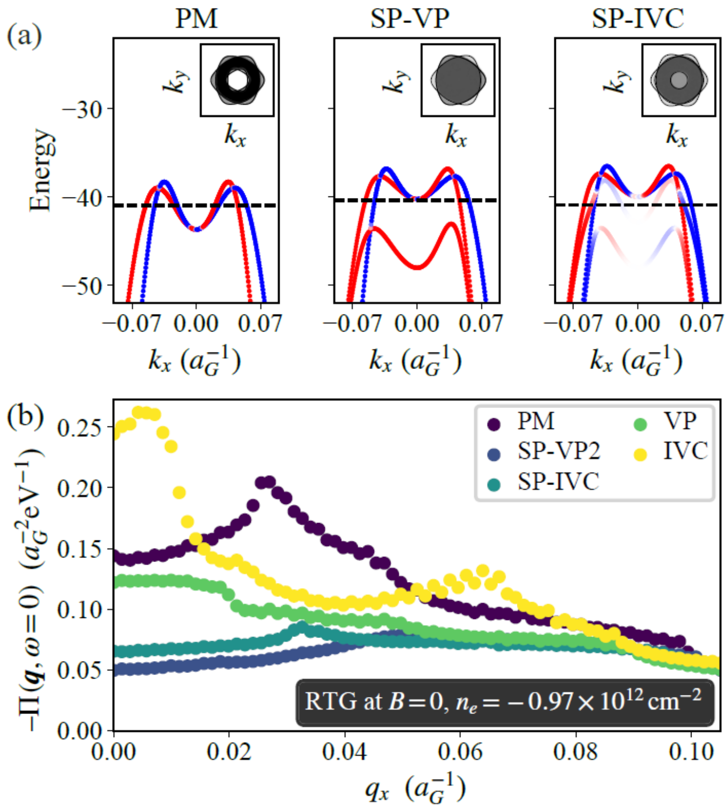

(c)

State X	$\delta E_{\mathrm{gap}}^{X} - \delta E_{\mathrm{gap}}^{\mathrm{PM}}$
SP-VP	1.3 meV
SP-IVC	1.2 meV
VP	0.6 meV
IVC	0.2 meV

Supplementary Figure 9. Static polarizability of spin-valley polarized states in rhombohedral trilayer graphene (RTG) at large displacement field $U_D = 30\mathrm{meV}$ and fixed electron density $n_e = -0.97 \times 10^{12} cm^{-2}$. (**a**) Mean-field bandstructures of the paramagnetic/symmetric state (PM), the spin and valley-polarized state (SP-VP), the spin-polarized intervalley-coherent (SP-IVC), valley-polarized (VP), and intervalley-coherent (IVC) state, where band colors indicate the valley expectation values (red: K, blue:K'). Insets illustrate the corresponding Fermi sea and surfaces (hue: hole occupation number). (**b**) Static polarizability vs q_x momentum for all candidate states, with stark differences that are associated with the phase space for particle-hole excitations on the Fermi sea at given $\boldsymbol{q}$. (**c**) Predicted bandgap renormalization in a nearby monolayer of WSe_2 when the system undergoes symmetry breaking from PM to symmetry-broken state, obtained by evaluating supplementary Eq. (1) using the static polarizability shown in panel (**b**).

Note that the candidate states were computed using self-consistent Fock mean-field theory using dielectric permittivity $\epsilon_r = 5.5$ and screening gate distance $d_{gate} = 50\mathrm{nm}$.

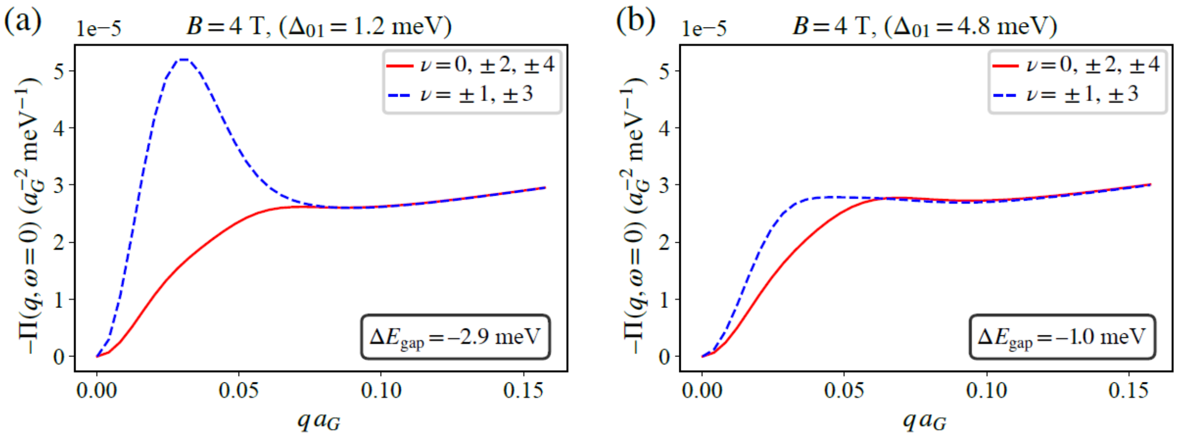

Supplementary Figure 10. Static polarizability of the Landau-quantized electronic spectrum of bernal bilayer graphene at magnetic field $B = 4$T and vanishing displacement field $U_D = 0$ at filling factors $\nu = -4, \dots, 4$. Due to interactions, the filling of the lowest Landau levels occurs flavor by flavor. The polarizability shows a strong even/odd dependence, due to additional interorbital transitions in each flavor sector between Landau level 0 and Landau level 1 that are separated by a gap Δ_{01}. We show two different cases for Δ_{01} in panel (**a**) and panel (**b**), respectively, and indicate the resulting difference in the proximity-induced WSe_2 bandshift between even and odd filling, $\Delta\, E_{gap} = \delta\, E_{gap}^{odd} - \delta\, E_{gap}^{even}$. In the results shown here, we imposed flavor polarization without performing self-consistent Hartree-Fock calculations.

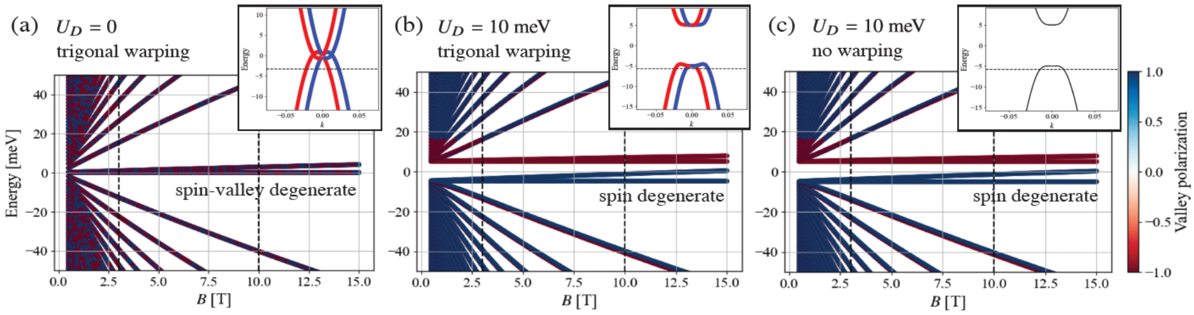

Supplementary Figure 11. Landau level spectra for bernal bilayer graphene (BBG) (sketched in inset) at large displacement fields. Insets show band structures at zero field $B = 0$. (**a**) Landau levels at zero displacement field, such that the lowest two (anomalous) landau levels are spin and valley degenerate. (**b**) Landau levels at finite displacement field, lifting the valley degeneracy in the anomalous Landau levels. (**c**) Landau levels when trigonal warping is neglected, illustrating that trigonal warping is negligible at large magnetic fields.

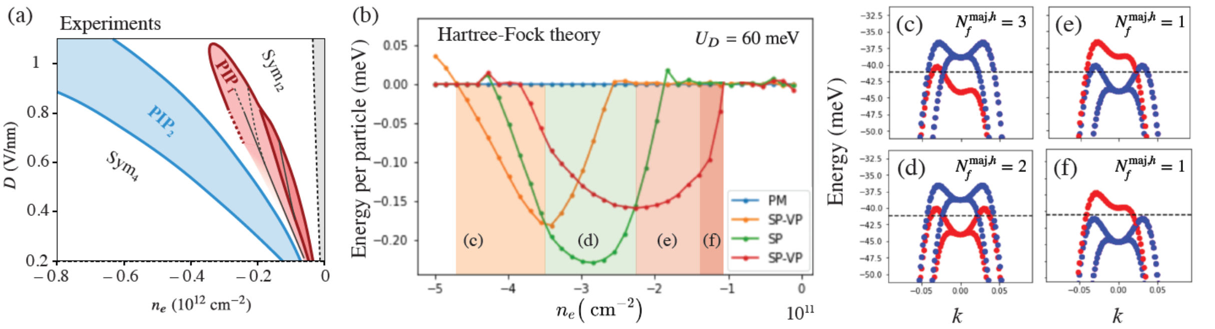

Supplementary Figure 12. Phase diagram of bernal bilayer graphene with partially and fully polarized magnetic phases (PIP, half-metal, quarter-metal). (**a**) Sketch of the phase diagram as observed experimentally in Ref.[11], with symmetric/paramagnetic phases (Sym_4, Sym_{12}) and partially polarized phases (PIP_1, PIP_2). (**b**) Hartree-Fock ground state energies of different types of paramagnetic and (c-f) spin-valley polarized states (PM, SP-VP, SP). The magnetic states differ by the number $N_f^{maj,h}$ of majorly hole-occupied flavors. (**c-f**) Examples for mean-field band structures of the magnetic states. Here, for simplicity, we do not include intervalley coherent states in the analysis. In panels (**b-f**), we used $U_D = 62\text{meV}$, $\epsilon_r = 4.4$, $d_{gate} = 40\text{nm}$.

Supplementary References

1. Hsu, W.-T. *et al.* Dielectric impact on exciton binding energy and quasiparticle bandgap in monolayer WS2 and WSe2. *2d Mater* **6**, 025028 (2019).

2. Hu, Q. *et al.* Observation of Rydberg moiré excitons. *Science (1979)* **380**, 1367–1372 (2023).

3. He, M. *et al.* Dynamically tunable moiré exciton Rydberg states in a monolayer semiconductor on twisted bilayer graphene. *Nat Mater* (2024) doi:10.1038/s41563-023-01713-y.

4. Xu, Y. *et al.* Creation of moiré bands in a monolayer semiconductor by spatially periodic dielectric screening. *Nat Mater* **20**, 645–649 (2021).

5. Bistritzer, R. & MacDonald, A. H. Moiré bands in twisted double-layer graphene. *Proceedings of the National Academy of Sciences* **108**, 12233–12237 (2011).

6. Hu, H., Bernevig, B. A. & Tsvelik, A. M. Kondo Lattice Model of Magic-Angle Twisted-Bilayer Graphene: Hund's Rule, Local-Moment Fluctuations, and Low-Energy Effective Theory. *Phys Rev Lett* **131**, 026502 (2023).

7. Koh, J. M., Alicea, J. & Lantagne-Hurtubise, É. Correlated phases in spin-orbit-coupled rhombohedral trilayer graphene. *Phys Rev B* **109**, 035113 (2024).

8. Szabó, A. L. & Roy, B. Competing orders and cascade of degeneracy lifting in doped Bernal bilayer graphene. *Phys Rev B* **105**, (2022).

9. Cao, Y. *et al.* Unconventional superconductivity in magic-angle graphene superlattices. *Nature* **556**, 43–50 (2018).

10. Park, J. M., Cao, Y., Watanabe, K., Taniguchi, T. & Jarillo-Herrero, P. Tunable strongly coupled superconductivity in magic-angle twisted trilayer graphene. *Nature* **590**, 249–255 (2021).

11. Zhou, H. *et al.* Isospin magnetism and spin-polarized superconductivity in Bernal bilayer graphene. *Science (1979)* **375**, 774–778 (2022).

12. de la Barrera, S. C. *et al.* Cascade of isospin phase transitions in Bernal-stacked bilayer graphene at zero magnetic field. *Nat Phys* **18**, 771–775 (2022).

13. Zhou, H. *et al.* Half- and quarter-metals in rhombohedral trilayer graphene. *Nature* **598**, 429–433 (2021).

14. Zhou, H., Xie, T., Taniguchi, T., Watanabe, K. & Young, A. F. Superconductivity in rhombohedral trilayer graphene. *Nature* **598**, 434–438 (2021).

15. Zondiner, U. *et al.* Cascade of phase transitions and Dirac revivals in magic-angle graphene. *Nature* **582**, 203–208 (2020).

16. Sharpe, A. L. *et al.* Emergent ferromagnetism near three-quarters filling in twisted bilayer graphene. *Science (1979)* **365**, 605–608 (2019).

17. Yu, J. *et al.* Correlated Hofstadter spectrum and flavour phase diagram in magic-angle twisted bilayer graphene. *Nat Phys* **18**, 825–831 (2022).

18. Saito, Y. *et al.* Isospin Pomeranchuk effect in twisted bilayer graphene. *Nature* **592**, 220–224 (2021).

19. Polshyn, H. *et al.* Large linear-in-temperature resistivity in twisted bilayer graphene. *Nat Phys* **15**, 1011–1016 (2019).

20. Hu, Q. *et al. Link between Cascade Transitions and Correlated Chern Insulators in Magic-Angle Twisted Bilayer Graphene*. (2024).

21. Holleis, L. *et al.* Fluctuating magnetism and Pomeranchuk effect in multilayer graphene. *Nature* (2025) doi:10.1038/s41586-025-08725-5.

22. Parto, K., Azzam, S. I., Banerjee, K. & Moody, G. Defect and strain engineering of monolayer WSe2 enables site-controlled single-photon emission up to 150 K. *Nat Commun* **12**, 3585 (2021).

23. Chen, Z., Luo, W., Liang, L., Ling, X. & Swan, A. K. Charge Separation in Monolayer WSe2 by Strain Engineering: Implications for Strain-Induced Diode Action. *ACS Appl Nano Mater* **5**, 15095–15101 (2022).

24. Schmidt, R. *et al.* Reversible uniaxial strain tuning in atomically thin WSe2. *2d Mater* **3**, 021011 (2016).

25. Nam, N. N. T. & Koshino, M. Lattice relaxation and energy band modulation in twisted bilayer graphene. *Phys Rev B* **96**, (2017).

26. Kwan, Y. H. *et al.* Kekulé Spiral Order at All Nonzero Integer Fillings in Twisted Bilayer Graphene. *Phys Rev X* **11**, (2021).

27. Raja, A. *et al.* Coulomb engineering of the bandgap and excitons in two-dimensional materials. *Nat Commun* **8**, 15251 (2017).

28. Popert, A. *et al.* Optical Sensing of Fractional Quantum Hall Effect in Graphene. *Nano Lett* **22**, 7363–7369 (2022).

29. Wolf, T., Jin, C. & MacDonald, A. H. Optical sensing of remote 2d-electron-fluid correlations. (2025), in preparation.

30. Wang, G. *et al.* Colloquium: Excitons in atomically thin transition metal dichalcogenides. *Rev Mod Phys* **90**, 021001 (2018).

31. Goerbig, M. O. Electronic properties of graphene in a strong magnetic field. *Rev Mod Phys* **83**, 1193–1243 (2011).

32. Zhang, F., Sahu, B., Min, H. & MacDonald, A. H. Band structure of ABC-stacked graphene trilayers. *Phys Rev B* **82**, 035409 (2010).

33. Koshino, M. & McCann, E. Trigonal warping and Berry's phase $n\pi$ in ABC-stacked multilayer graphene. *Phys Rev B* **80**, 165409 (2009).

34. Lu, Z. *et al.* Fractional quantum anomalous Hall effect in multilayer graphene. *Nature* **626**, 759–764 (2024).

35. Han, T. *et al.* Correlated insulator and Chern insulators in pentalayer rhombohedral-stacked graphene. *Nat Nanotechnol* **19**, 181–187 (2024).

36. Zhang, Y. *et al.* Enhanced superconductivity in spin–orbit proximitized bilayer graphene. *Nature* **613**, 268–273 (2023).
