# Supplementary material for: Optical imaging of flavor order in flat band graphene
Source: Nat Commun. 2025 Jul 1;16:5555. doi: 10.1038/s41467-025-60675-8 (PMC12218313; doi:10.1038/s41467-025-60675-8)
Supplement: Supplementary file 2 — Description of Additional Supplementary Files [file 41467_2025_60675_MOESM2_ESM.pdf]

Description of Additional Supplementary Files for "Optical Imaging of Flavor Order in Flat Band Graphene"

Authors: Tian Xie[1], Tobias M. Wolf[2], Siyuan Xu[1], Zhiyuan Cui[1], Richen Xiong[1], Yunbo Ou[3], Patrick Hays[3], Ludwig F Holleis[1], Yi Guo[1], Owen I Sheekey[1], Caitlin Patterson[1], Trevor Arp[1], Kenji Watanabe[4], Takashi Taniguchi[5], Seth Ariel Tongay[3], Andrea F Young[1], Allan H. MacDonald[2]*, Chenhao Jin[1]*

Affiliations:

[1]Department of Physics, University of California at Santa Barbara, Santa Barbara, CA, 93116, USA

[2]Department of Physics, University of Texas at Austin, Austin, TX, 78712, USA

[3]Materials Science and Engineering Program, School of Engineering for Matter, Transport, and Energy, Arizona State University, Tempe, Arizona 85287, USA

[4]Research Center for Electronic and Optical Materials, National Institute for Materials Science, 1-1 Namiki, Tsukuba 305-0044, Japan

[5]Research Center for Materials Nanoarchitectonics, National Institute for Materials Science, 1-1 Namiki, Tsukuba 305-0044, Japan

* Corresponding author. Email: macd@physics.utexas.edu, jinchenhao@ucsb.edu

Contents:

Description of Additional Supplementary Movies

Supplementary Movies

Supplementary Movie 1: Wide-field imaging of MATBG device D5 with probe energy 698.5nm at different doping from $0.91\times10^{12}cm^{-2}$ to $1.38\times10^{12}cm^{-2}$

Supplementary Movie 2: Wide-field imaging of MATBG device D5 with probe energy 700.4nm at different doping from $2.33\times10^{12}cm^{-2}$ to $3.34\times10^{12}cm^{-2}$
